# Equipping community health workers with digital tools for pandemic response in LMICs

**DOI:** 10.1186/s13690-020-00513-z

**Published:** 2021-01-04

**Authors:** Anam Shahil Feroz, Adeel Khoja, Sarah Saleem

**Affiliations:** 1 Department of Community Health Sciences, The Aga Khan University, Stadium Road, PO Box 3500, Karachi, 74800 Pakistan; 2 Institute of Health Policy, Management, and Evaluation, University of Toronto , Toronto, Ontario Canada; 3 Department of Medicine, The Aga Khan University, Stadium Road, PO Box 3500, Karachi, 74800 Pakistan; 4 Faculty of Health and Medical Sciences Adelaide Medical School, The University of Adelaide, Adelaide, South Australia 5005 Australia

**Keywords:** Community health workers (CHWs), Digital tools, Covid-19, Low-middle-income countries

## Abstract

**Background:**

Community health workers (CHWs) are well-positioned to play a pivotal role in fighting the pandemic at the community level. The Covid-19 outbreak has led to a lot of stress and anxiety among CHWs as they are expected to perform pandemic related tasks along with the delivery of essential healthcare services. In addition, movement restrictions, lockdowns, social distancing, and lack of protective gear have significantly affected CHWs’ routine workflow and performance. To optimize CHWs’ functioning, there is a renewed interest in supporting CHWs with digital technology to ensure an appropriate pandemic response.

**Discussion:**

The current situation has necessitated the use of digital tools for the delivery of Covid-19 related tasks and other essential healthcare services at the community level. Evidence suggests that there has been a significant digital transformation to support CHWs in these critical times such as remote data collection and health assessments, the use of short message service and voice message for health education, use of digital megaphones for encouraging behavior change, and digital contract tracing. A few LMICs such as Uganda and Ethiopia have been successful in operationalizing digital tools to optimize CHWs’ functioning for Covid-19 tasks and other essential health services.

**Conclusion:**

Yet, in most LMICs, there are some challenges concerning the feasibility and acceptability of using digital tools for CHWs during the Covid-19 pandemic. In most cases, CHWs find it difficult to adopt and use digital health solutions due to lack of training on new digital tools, weak technical support, issues of internet connectivity, and other administrative related challenges. To address these challenges, engaging governments would be essential for training CHWs on user-friendly digital health solutions to improve routine workflow of CHWs during the Covid-19 pandemic.

## Background

Over 15,415,727 Coronavirus disease (Covid-19) cases and 631,164 deaths have been reported, as of July 23, 2020 [1]. The pandemic has disproportionately affected the poor and vulnerable and has overwhelmed the health systems of LMICs. Investment is needed at all levels of the health system, and especially at the community level whereby community health workers (CHWs) are poised to play a pivotal role in fighting the pandemic. CHWs in low-and-middle-income countries (LMICs) are considered as an essential component of the primary health care (PHC) system as they primarily act as a liaison between local communities and the primary healthcare facilities. CHWs are a tremendous asset to health systems because of their community-rooted credibility and understanding. These CHWs deliver a variety of promotive, preventive, and curative, and rehabilitative healthcare services in underprivileged areas. The health information and advice offered by CHWs is highly appreciated and recognized by the population as these workers are often the first and the only point of contact for the community, especially for pregnant women, newborns, and under 5 children. In LMICs, CHWs have been significantly engaged in numerous PHC initiatives and vertical programs instigated by the public health department, non-governmental organizations (NGOs), and donor agencies. These initiatives include maternal, newborn, and child health program (MNCH), nutrition program, vaccination program, communicable disease control program, health emergency response activities, disease surveillance, etc. Thus, there is a wide scope of services offered by the CHWs to the community, ranging from the provision of safe delivery, and counseling on breastfeeding, management of uncomplicated childhood illnesses, to preventive health education on communicable and non-communicable diseases (NCDs). Evidence suggests that the healthcare services provided by these workers have significantly contributed to the decline in maternal and child morbidity and mortality rates and have also assisted in decreasing the burden of communicable and non-communicable diseases. Most CHWs in LMICs provide primary healthcare services and education to communities through home-to-home visits, and group training [1].

However, in the wake of Covid-19, the CHWs’ usual job duties have been significantly affected for a variety of reasons including movement restrictions, lockdowns, and social distancing. The Covid-19 outbreak has led to a lot of stress and anxiety among CHWs [2]. Some workers are concerned that as a result of door-to-door community visits, they might get infected and transmit the virus to their family members. A few CHWs are apprehensive of becoming vectors of spreading COVID-19 in communities. Lack of personal protective equipment (PPE) has also restricted many CHWs to reach out to families in need [3]. In some LMICs, CHWs have received guidelines from the health system to either stay home, stop working, and cease providing essential health-care services to their communities. Despite being a vital part of the Covid-19 pandemic response, CHWs in LMICs are not well-supported and equipped with resources to contain the spread of Covid-19. This is the time when health systems of LMICs should focus on strengthening the role of CHWs for health education, case detection, and linking people to care to fight against the Covid-19 pandemic. An apparent and accessible solution is coupling efforts of CHWs with digital technology to ensure an appropriate pandemic response. The Covid-19 pandemic has resulted in a greater demand for digital health innovations at the community health level.

## Main text

### Potential pandemic-related tasks for CHWs to reduce the devastating impact of Covid-19

In some LMICs, CHWs are playing a huge role in providing essential health care services and Covid-19 related healthcare to the communities. This is especially needed in the face of infectious disease outbreaks when there are huge shortages of healthcare professionals and the immense psychological and emotional burden on healthcare providers. As the number of Covid-19 cases in LMICs continues to rise, the global health community is debating on what roles CHWs could and should play in the prevention, detection, and management of Covid-19 cases [3]. To create awareness among communities about Covid-19, CHWs serve as a trusted communicator to give culturally appropriate information on hand hygiene, sanitation, and social distancing. In addition, CHWs have a huge role in dispelling misconceptions and counter the social stigma associated with Covid-19. CHWs promote skills for behavioral change by setting up handwashing stations and encouraging mask-wearing and physical distancing. However, when social distancing is not possible, early detection and isolation are central to stop the virus spread [4]. To detect Covid-19 cases, CHWs are expected to identify early signs and symptoms of Covid-19 disease, supporting isolation, monitoring individuals who are self-isolating, and referring more severe cases to the healthcare facilities. This requires CHWs to perform outreach services by coordinating with the communities and specialized physicians to ensure contact-tracing of Covid-19 cases [5]. Liberia is preparing CHWs to assist with case detection and South Africa is involving CHWs in mass screening and testing through the COVID Home Visits program [4]. Alongside detection, CHWs are expected to manage Covid-19 cases by providing direct services and treatment for mild symptoms. CHWs can play a role in distributing commodities such as, food items and other essential household products to those who are self-isolating. With the prevention, detection, and management of Covid-19 cases, CHWs are also rapidly trained to deliver the basic mental health services to the community people to address issues of stress, anxiety, and depression, which are rising because of COVID-19 [2]. At the same time, CHWs are expected to provide other essential services including antenatal and postnatal care, child immunization, and primary treatment of pneumonia, malaria, tuberculosis, and diarrhea. Assigning these new Covid-19 related tasks to CHWs, within the scope of existing roles, pose the question of whether these additional tasks will produce significant population health benefits and outweigh the risks posed to CHWs. As it makes little sense to divert all CHWs for pandemic preparedness and response at the expense of other essential services, public health departments, NGOs and social enterprises operating CHW programs need to strike the right balance, between Covid-19 related tasks and other essential services [5].

### Harnessing digital technology to support CHWs in the Covid-19 response

A plausible way to optimize CHWs’ functioning during this pandemic is to pair the efforts of CHWs with digital tools to prevent their exposure to Covid-19. CHWs equipped with digital tools can serve as a valuable lifeline in the Covid-19 response for information sharing, communication, training, surveillance, and decision support. The current situation has necessitated innovation for the delivery of Covid-19 related tasks and other essential healthcare services at the community level. Evidence suggests that there has been a significant digital transformation to support CHWs in these critical times such as remote data collection and health assessments, use of short message service (SMS) and voice message for health education, use of digital megaphones for encouraging behavior change, digital contract tracing, etc. [6]. Digital health technology has been reportedly used for supporting and monitoring CHW programs during the Covid-19 pandemic. In Ethiopia, CHWs are raising awareness on Covid-19 using megaphones and by playing audio messages in the local language. In Uganda, CHWs require communication materials such as megaphones to be able to provide health education and skills for behavioral change. In Vietnam, the Ministry of Health has piloted a telemedicine platform to reduce direct contact of CHWs with community patients, decreasing the risk of infection for community-based health workers, in the context of the Covid-19 pandemic. In Mozambique [7], upSCALE digital platform has been adapted to support CHWs to respond to COVID-19. The CHWs in Mozambique utilize upSCALE digital platform to provide basic healthcare and conduct health promotion activities [8]. Several digital tools have been operationalized to optimize CHWs’ functioning for Covid-19 tasks and other essential health services. These include Living Goods’ Smart Health app, DiMagi’s CommCare, mHero, and Medic Mobile’s Community Health Toolkit [6, 9]. Living Goods has integrated Covid-19 protocol into the SmartHealth app and working to develop an SMS messaging platform to help equip CHWs with the most up-to-date tools to fight COVID in their communities [6]. Dimagi’s CommCare is an offline-first, open-source mobile data collection and service delivery platform that helps CHWs in every phase of effective COVID-19 response - from screening and contact tracing to patient monitoring and post-care support [10]. mHero, a mobile phone-based communication system, was created by IntraHealth International and UNICEF in 2014 in Liberia for an Ebola outbreak [11]. It operates on a basic talk-and-text pattern and no smartphone or tablet is mandatory and at present, it is being used to update healthcare workers regarding Covid-19. Literature suggests that the data that CHWs are providing through digital tools is invaluable not just for tracking and intervening against Covid-19, but also to monitor the disruption of services which is a huge concern in managing high-risk pregnancies, providing essential antenatal and postnatal care, and supporting TB patients at risk of discontinuing their treatment [9].

### Preparedness of CHWs to use digital health for pandemic response

However, digital health tools aren’t a silver bullet, to fix everything including the delivery of Covid-19 related healthcare services or ensuring the long-term delivery of other essential health services. Challenges still exist in building the capacity of CHWs to use digital tools and interpret data collected. The lack of digital skills among CHWs is not unique to the health system in LMICs. CHWs encounter unique challenges when adopting and using digital health solutions for health service delivery. These include lack of CHWs training on new digital tools, weak technical support, issues of internet connectivity, and other administrative related challenges. The 2019 Global Digital Health Index reports that most LMICs need digital health training to meet current demand, and digital considerations are yet to be properly reflected. Currently, only a few LMICs show promising results, in creating specialized digital health workers, including Bangladesh, Chile, Ethiopia, Kuwait, Malaysia, Peru, Portugal, Sri Lanka, Thailand, and Uganda. Utilizing existing digital health technology systems and expanding and adapting it to evolving healthcare needs, would be critical for managing the current pandemic [12]. Additionally, factors such as age, education level, years of experience, and technology proficiency levels will also affect CHWs’ readiness to adopt new digital health solutions for Covid-19. A systematic review conducted by Agarwal et al., reported that front line health workers (FHWs) with the low level of literacy often find it difficult to adapt to new digital health interventions. Therefore, user-friendly digital health solutions should be developed and integrated into the routine workflow of CHWs for pandemic preparedness and response [1]. Training of CHWs on basic technological skills such as the use of digital megaphones and robocalls for health education, SMS for data collection, and voice call for a consultation, will help accelerate the response to the Covid-19 pandemic [7]. Engaging governments on the approach for training existing CHWs to prevent, detect, and manage Covid-19 cases, would be useful in ensuring better community health [13].

## Conclusion

In the wake of infectious disease outbreaks, CHWs are playing a huge role in providing essential health care services and Covid-19 related healthcare to the communities. CHWs are overburdened as they are expected to accomplish more although they are not getting the required support to perform their duties well, such as training, remuneration, protective gear, etc. There is an ample evidence base suggesting that the use of digital interventions is essential to optimize the functioning of CHWs during the Covid-19 pandemic in LMICs. The digital tools have been used by CHWs in various settings for the prevention, detection, and management of Covid-19 cases. In LMICs, equipping CHWs with digital tools will ensure that CHWs have an organized workflow for carrying out Covid 19-related tasks and other essential duties. The integration of digital health will help optimize the functioning of CHWs in a number of ways including health education, contract tracing, and remote supervision. There are some challenges concerning the feasibility and acceptability of using digital tools for CHWs during the Covid-19 pandemic. It is anticipated that CHWs will encounter unique challenges when adopting and using digital health solutions for the delivery of Covid-19 tasks and other essential healthcare services. Given the gaps in the literature, future research efforts and policy dialogue should be directed to explore health system readiness for training CHWs on basic digital health solutions to improve CHWs’ workflow and performance for Covid-19 response.

## Data Availability

Not applicable.

## Abbreviations

CHWs: Community health workers
Covid-19: Coronavirus disease
FHWs: Front line health workers
LMICs: Low-and-middle-income countries
MNCH: Maternal, newborn and child health program
NCDs: Non-communicable diseases
NGOs: Non-governmental organizations
PPEs: Personal protective equipment
PHC: Primary health care
SMS: Short message service
